# Expansion of the Gateway MultiSite Recombination Cloning Toolkit

**DOI:** 10.1371/journal.pone.0077724

**Published:** 2013-10-18

**Authors:** Harold K. Shearin, Alisa R. Dvarishkis, Craig D. Kozeluh, R. Steven Stowers

**Affiliations:** Department of Cell Biology and Neuroscience, Montana State University, Bozeman, Montana, United States of America; Columbia University, United States of America

## Abstract

Precise manipulation of transgene expression in genetic model organisms has led to advances in understanding fundamental mechanisms of development, physiology, and genetic disease. Transgene construction is, however, a precondition of transgene expression, and often limits the rate of experimental progress. Here we report an expansion of the modular Gateway MultiSite recombination-cloning platform for high efficiency transgene assembly. The expansion includes two additional destination vectors and entry clones for the LexA binary transcription system, among others. These new tools enhance the expression levels possible with Gateway MultiSite generated transgenes and make possible the generation of LexA drivers and reporters with Gateway MultiSite cloning. *In vivo* data from transgenic *Drosophila* functionally validating each novel component are presented and include neuronal LexA drivers, LexAop2 red and green fluorescent synaptic vesicle reporters, TDC2 and TRH LexA, GAL4, and QF drivers, and LexAop2, UAS, and QUAS channelrhodopsin2 T159C reporters.

## Introduction

 Transgene expression is an integral component of experimental approaches in genetic model organisms for addressing a wide variety of biological problems. Sophisticated transgene expression experiments may require precise control of the timing, location, and level of transgene expression as well as independent control of expression of multiple transgenes simultaneously. A prerequisite for experiments involving transgene expression is, however, the construction of transgenes using recombinant DNA methods. Producing these transgenes is often a cumbersome and time-consuming process that has limited the rate of experimental progress due to the inefficiency of restriction enzyme-based cloning methods. Previously, we reported the development of a starter toolkit for Gateway MultiSite recombination cloning that includes a detailed description of the methodology by which two, three, or four fragments can be simultaneously recombination-cloned into a destination vector to generate expression clones [1]. This recombinase-based cloning methodology is efficient and reliable, and represents a significant advance over restriction-enzyme based cloning in terms of the ease and speed with which transgenic constructs can be produced. The Gateway MultiSite starter toolkit previously reported included a single destination vector and entry clones for the GAL4 and Q binary transcription systems, among others. 

 In this work we report an expansion of the Gateway MultiSite cloning platform including two new destination vectors that offer enhanced levels of transgene expression, entry clones for the LexA binary transcription system [2,3], and several additional entry clones. The new destination vectors provide flexibility in dictating appropriate transgene expression levels as required for specific experiments. Entry clones for the LexA system allow Gateway MultiSite construction of LexA drivers and reporters for experiments requiring independent control of expression of more than one transgene simultaneously. The functionality of these new tools for Gateway MultiSite cloning is demonstrated *in vivo* using the *Drosophila* model system. The fly strains demonstrating the functionality of these new Gateway MultiSite cloning tools include neuronal LexA drivers, LexAop2 red and green fluorescent synaptic vesicle reporters, tyrosine decarboxylase 2 (TDC2) and tryptophan hydroxylase (TRH) LexA, GAL4, and QF drivers, and LexAop2, UAS, and QUAS channelrhodopsin2 T159C reporters. 

## Results

### New Gateway MultiSite destination vectors with enhanced expression

**Figure 1:**
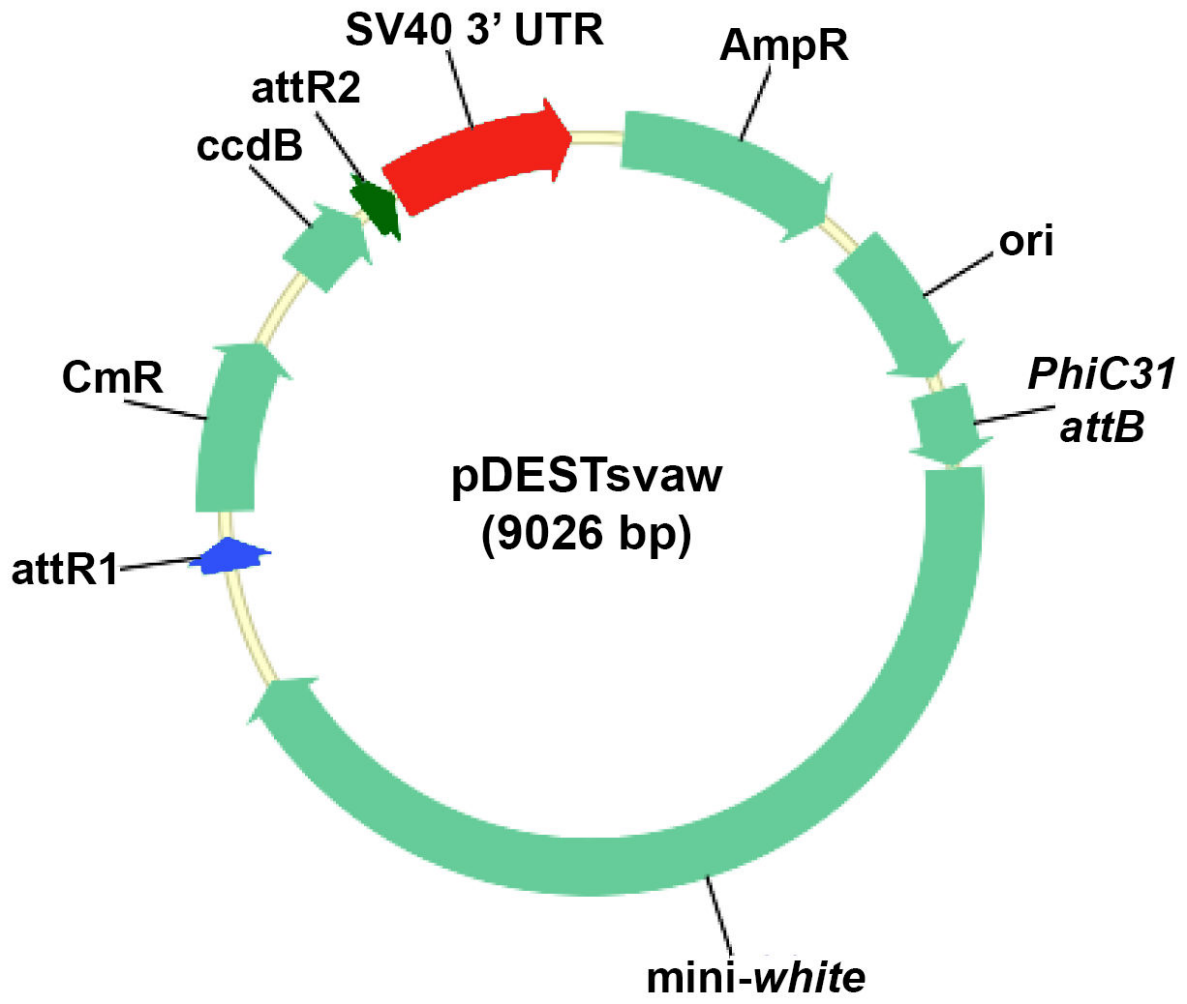
The *Drosophila* Gateway MultiSite destination vector pDESTsvaw. The pDESTsvaw destination vector contains a *PhiC31* attB site for *PhiC31* integrase-mediated site-specific transgenesis, a mini-white transformation marker, a Gateway cassette including flanking attR1 (blue) and attR2 (dark green) recombination sites, and an *SV40* 3’ UTR (red). The pDESTp10aw destination vector described in the text is identical except the *SV40* 3’ UTR has been replaced by the *p10* 3’ UTR. Both vectors are compatible with two-, three-, and four-fragment Gateway MultiSite cloning. CmR-chloramphenicol resistance, AmpR-ampicillin resistance, svaw-*SV40*, *PhiC31* attB, white.

 With the goal of enhancing transgene expression levels from expression clones generated via Gateway MultiSite cloning, two new *Drosophila* Gateway MultiSite destination vectors were constructed. The previously introduced *Drosophila* Gateway MultiSite destination vector pDESThaw utilized the *hsp70* 3’ UTR [1]. A recent report characterizing transgene expression levels for several different 3’ UTRs [4] suggested higher levels of transgene expression would be possible with Gateway MultiSite cloning if Gateway MultiSite destination vectors with alternative 3’ UTRs were developed. Two Gateway MultiSite destination vectors were assembled that utilize the *SV40* (pDESTsvaw) (Figure 1) or *p10* (pDESTp10aw) 3’UTRs [4]. These two destination vectors are identical except for the 3’ UTR and, like pDESThaw, contain a mini-white transgenesis marker, a *PhiC31* attB site for *PhiC31* recombinase-mediated transgenesis, and an attR1/attR2 Gateway cassette. 

**Figure 2:**
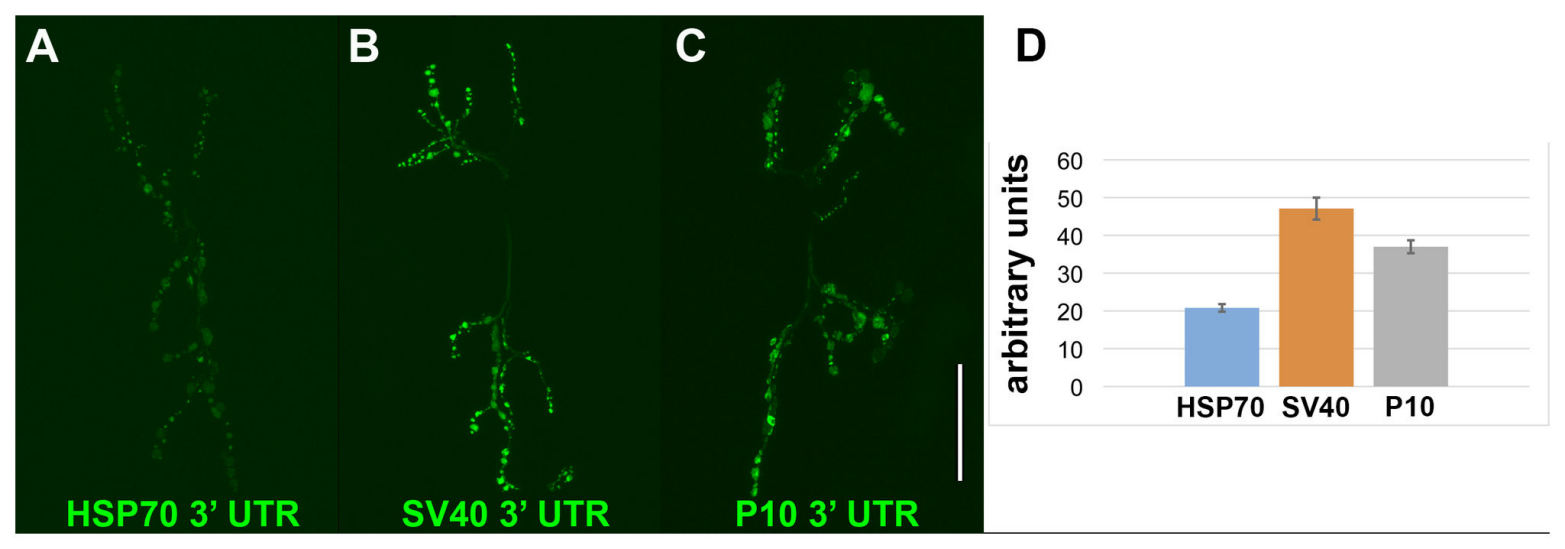
Quantitative comparison of motor neuron GFP-Rab3 expression levels in Gateway destination vectors with *hsp70*, *SV40*, and *p10* 3’ untranslated regions. Representative confocal images of direct GFP fluorescence for (A) pDESThaw (*hsp70* 3’ UTR); (B) pDEST svaw (*SV40* 3’ UTR), and (C) pDESTp10aw (*p10* 3’ UTR). All animals are of genotype *yw*; *vGlut-LexA*/*+*; *13XLexAop2-GFP-Rab3*/*+*. All three reporter transgenes were targeted to the VK00027 landing site to eliminate position effects as a source of expression level variability. The imaged neuromuscular junctions connected to muscles 6/7 on segment A4. (D) Quantitation of data from A-C in arbitrary digitizer units. pDESThaw 20.8 +/- 1.01 (SEM), pDESTsvaw 47.09 +/- 2.91, pDESTP10aw 36.99 +/- 1.72. SEM-standard error of the mean. Scale bar: 50μm.

 To assess the relative levels of transgene expression from the three Gateway MultiSite destination vectors, expression clones for each were generated that contain 13XLexAop2-GFPRab3. The L1-13XLexAop2-L4 entry clone used in these constructs is newly reported here and is described below, as is the construction of 13LexAop2-GFPRab3. To eliminate variability in expression levels due to position effects, all three constructs were inserted into the VK00027 landing site [5]. To quantitate expression levels, fly strains for each of the three 13XLexAop2-GFPRab3 constructs were crossed to a vGlut-LexA driver and the neuromuscular junctions innervating muscles 6 and 7, segment A4, of wandering third instar larva were imaged on a confocal microscope using identical settings. Representative images for each genotype are shown in Figures 2A-C. Three-dimensional reconstructions were generated and fluorescence intensities were quantitated using Imaris software. The quantitation results are shown in the bar graph in Figure 2D. The pDESTsvaw and pDESTp10aw destination vectors exhibited 2.26 and 1.78-fold higher expression levels, respectively, than pDESThaw. 

**Figure 3:**
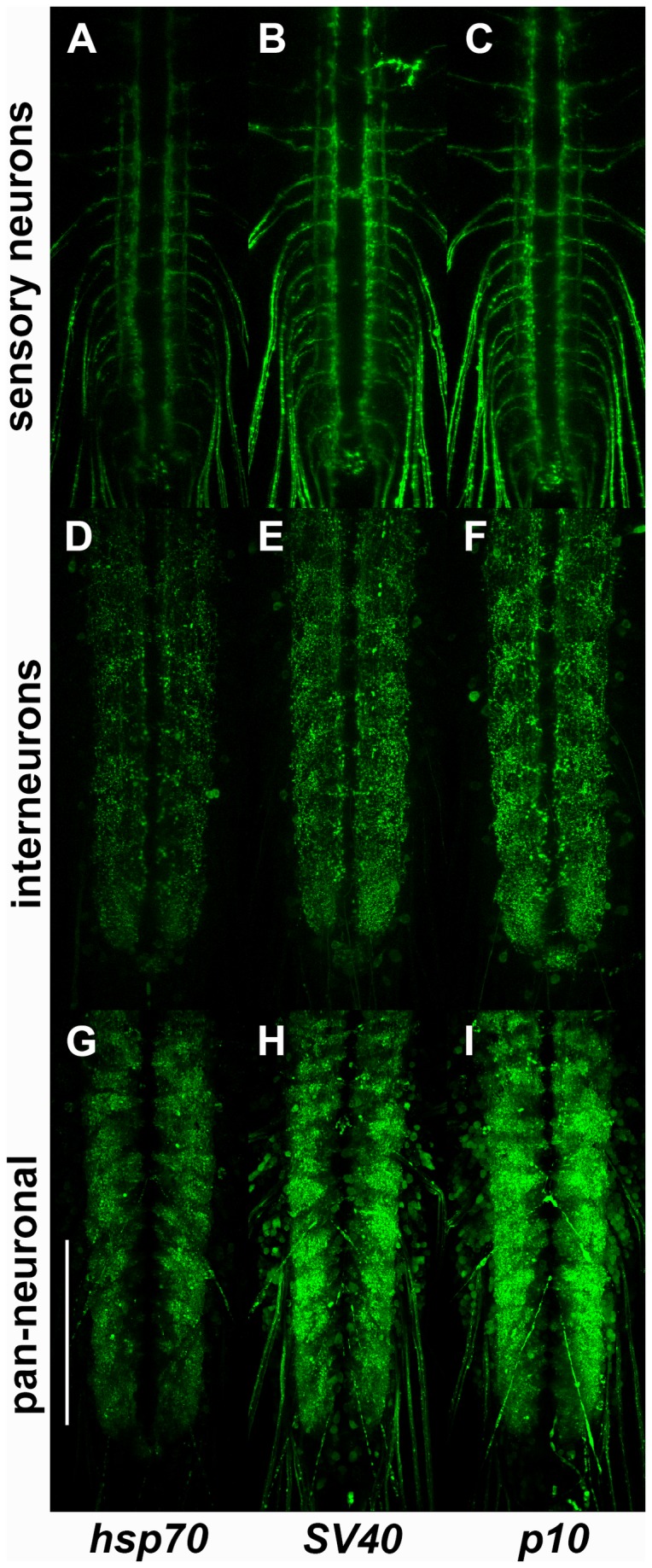
Qualitative comparison of sensory neuron, interneuron, and pan-neuronal GFP-Rab3 expression levels in Gateway destination vectors with *hsp70*, *SV40*, and *p10* 3’ untranslated regions. Representative confocal images of direct GFP fluorescence for (A, D, G) pDESThaw (*hsp70* 3’ UTR); (B, E, H) pDESTsvaw (*SV40* 3’ UTR), and (C, F, I) pDESTp10aw (*p10* 3’ UTR). (A-C) *yw*; *nompC-LexA*/*+*; *13XLexAop2-GFP-Rab3*/*+*; (D-F) *yw*; *TRH-LexA*/*+*; *13XLexAop2-GFP-Rab3*/*+*; (G-I) *yw*; *n-syb-LexA*/*+*; *13XLexAop2-GFP-Rab3*/*+*. All three reporter transgenes were targeted to the VK00027 landing site to eliminate position effects as a source of expression level variability. Expression of GFP-Rab3 with the *hsp70* 3’ UTR exhibits consistently lower expression levels than GFP-Rab3 with the *SV40* or *p10* 3’ UTRs which show comparable expression levels. All images for a given driver were acquired using identical confocal settings. Scale bar 100μm.

 To determine if the quantitative results obtained in motor neurons generalize to other neuron types, similar qualitative experiments were performed using the sensory neuron driver nompC-LexA, the interneuron driver TRH-LexA, and the pan-neuronal driver n-syb-LexA. The construction of these three drivers is described below. With all three drivers, the expression of GFP-Rab3 from pDESThaw (Figures 3A, D, G) was noticeably less than that of GFP-Rab3 from pDESTsvaw (Figures 3B, E, H) or pDESTp10aw (Figures 3C, F, I), which were comparable. Although minor variations were observed, the trend in expression levels of GFP-Rab3 for sensory neurons, interneurons, and with pan-neuronal expression, was in general agreement with the quantitative results obtained for motor neurons. This suggests the effect on expression levels of each of the three UTRs does not vary significantly across neuronal subtypes. Overall, these quantitative and qualitative results demonstrate the new pDESTsvaw and pDESTp10aw destination vectors allow enhanced levels of transgene expression for Gateway MultiSite generated transgenes.

 The complete nucleotide sequences of the pDESTsvaw and pDESTp10aw destination vectors, as well as all entry clones described in this report, can be found at www.gatewaymultisite.org. A complete list of all entry clones and fly strains newly described herein can be found in Tables 1 and 2, respectively.

**Table 1:** Entry clones.

**Entry clones**	**Insert size [bp]**
R4-LexAp65-R3	1448
L5-LexAp65-L2	1452
L1-13XLexAop2-L4	666
L1-13XLexAop2-R5	663
L1-*TDC2*-5'Reg-L4	4145
L3-*TDC2*-3'Reg-L2	4147
L1-*n-syb*-R5	878
L1-*TRH*-R5	1299
L5-2XHA-Rab3-L2	754
L5-Chr2 T159C-HA-L2	987

**Table 2:** Expression clones/Fly stocks.

**Construct**	**Insertion site[s]**	**Vector**
13XLexAop2-GFP-Rab3	VK00027	pDESThaw
13XLexAop2-GFP-Rab3	VK00027	pDESTsvaw
13XLexAop2-GFP-Rab3	VK00027	pDESTP10aw
nompC-LexAp65	VK00027/VK00018	pDESTP10aw
TDC2-LexAp65	attP40	pDESTsvaw
TDC2-GAL4	attP2	pDESThaw
TDC2-QF	VK00027/attP33	pDESThaw
iav-LexAp65	VK00013	pDESTP10aw
n-syb-LexAp65	VK00018	pDESTsvaw
TRH-LexAp65	attP40	pDESTsvaw
TRH-GAL4	attP2	pDESThaw
TRH-QF	VK00027/attP33	pDESThaw
13XLexAop2-mCherry-Rab3	VK00018	pDESTsvaw
13XLexAop2-n-syb-4XmCherry	VK00027/attP40	pDESTsvaw
13XLexAop2-2XHA-Rab3	VK00018	pDESTsvaw
20XUAS-2XHA-Rab3	VK00018	pDESTsvaw
10XQUAS-2XHA-Rab3	VK00018	pDESTsvaw
13XLexAop2-Chr2T159C-HA	VK00013	pDESTsvaw
20XUAS-Chr2T159C-HA	VK00018/VK00027	pDESTsvaw
10XQUAS-Chr2T159C-HA	VK00018/VK00027	pDESTsvaw

### Functional LexAp65 Gateway MultiSite entry clones

 L5-LexAp65-L2 (compatible with two-fragment Gateway MultiSite cloning) and R4-LexAp65-R3 (compatible with three- and four-fragment Gateway MultiSite recombination cloning) entry clones were constructed. To assess the functionality, of the L5-LexAp65-L2 entry clone, it was combined with L1-iav 5’ Reg-R5 or L1-n-syb 5’ Reg-R5 in separate two-fragment LR reactions to generate the expression clones iav-LexAp65 and n-syb-LexAp65. 

**Figure 4:**
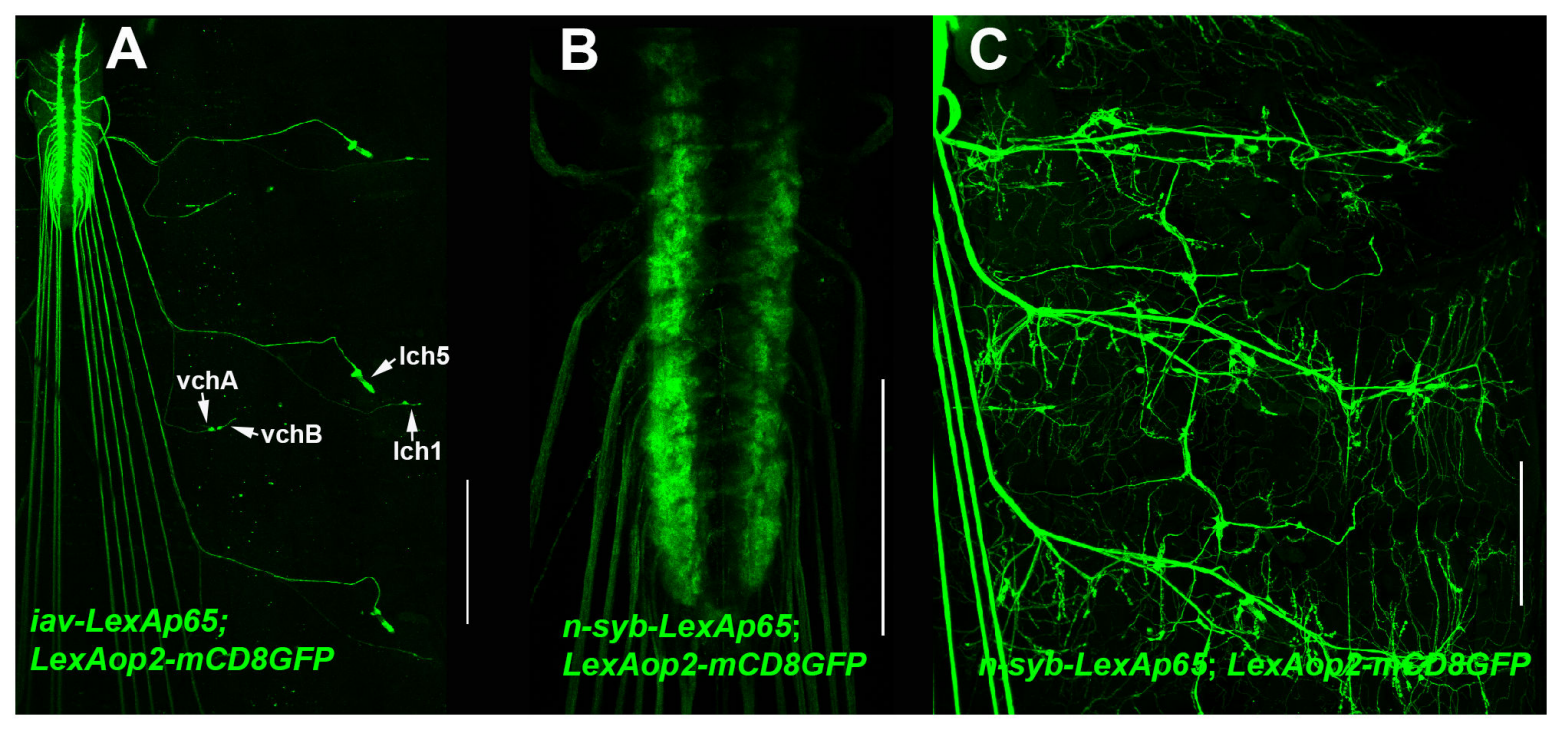
Functional demonstration of the L1-LexAp65-R5 Gateway MultiSite entry clone. Representative confocal images of third instar larval expression of (A) *yw*; *iav-LexAp65*/*LexAop-mCD8GFP*; (B-C) *yw*; *n-syb-LexAp65*/*+*; *LexAop-mCD8GFP*/*+*. iav-LexAp65 expresses exclusively in chordotonal organs. n-syb-LexAp65 expresses broadly in the CNS (B) and sensory and motor neurons (C). Anti-GFP was used as the primary antibody for (A) and (C). Direct GFP fluorescence was used for imaging (B). Scale bars: (A) 200μm, (B) 100μm, (C) 200μm.

 The third instar larval expression pattern of iav-LexAp65 is shown in Figure 4A using the reporter LexAop2-mCD8GFP. Similar to the previously reported iav-GAL4 and iav-QF drivers [1], iav-LexAp65 expresses exclusively in the vchA, vchB, lch5, and lch1 chordotonal organs (arrows). This chordotonal organ-specific expression pattern is consistent with the known role of the *inactive* (*iav*) gene in proprioception and hearing [6,7]. 

 The third instar larval expression pattern of n-syb-LexAp65 is shown in Figures 4B and C using the reporter LexAop2-mCD8GFP. n-syb-LexAp65 expresses extensively in the nervous system as indicated by broad expression in both the ventral nerve cord (Figure 4B) and in the peripheral body wall (Figure 4C) where abundant neuromuscular junction and sensory neuron expression is observed. The broad neuronal expression pattern of n-syb-LexAp65 is consistent with the known role of *neuronal-synaptobrevin* (*n-syb*) as a synaptic vesicle-specific SNARE protein required for synaptic vesicle fusion with the plasma membrane [8,9]. These results demonstrate the functionality of the L5-LexAp65-L2 and L1-n-syb 5’ Reg-R5 entry clones.

**Figure 5:**
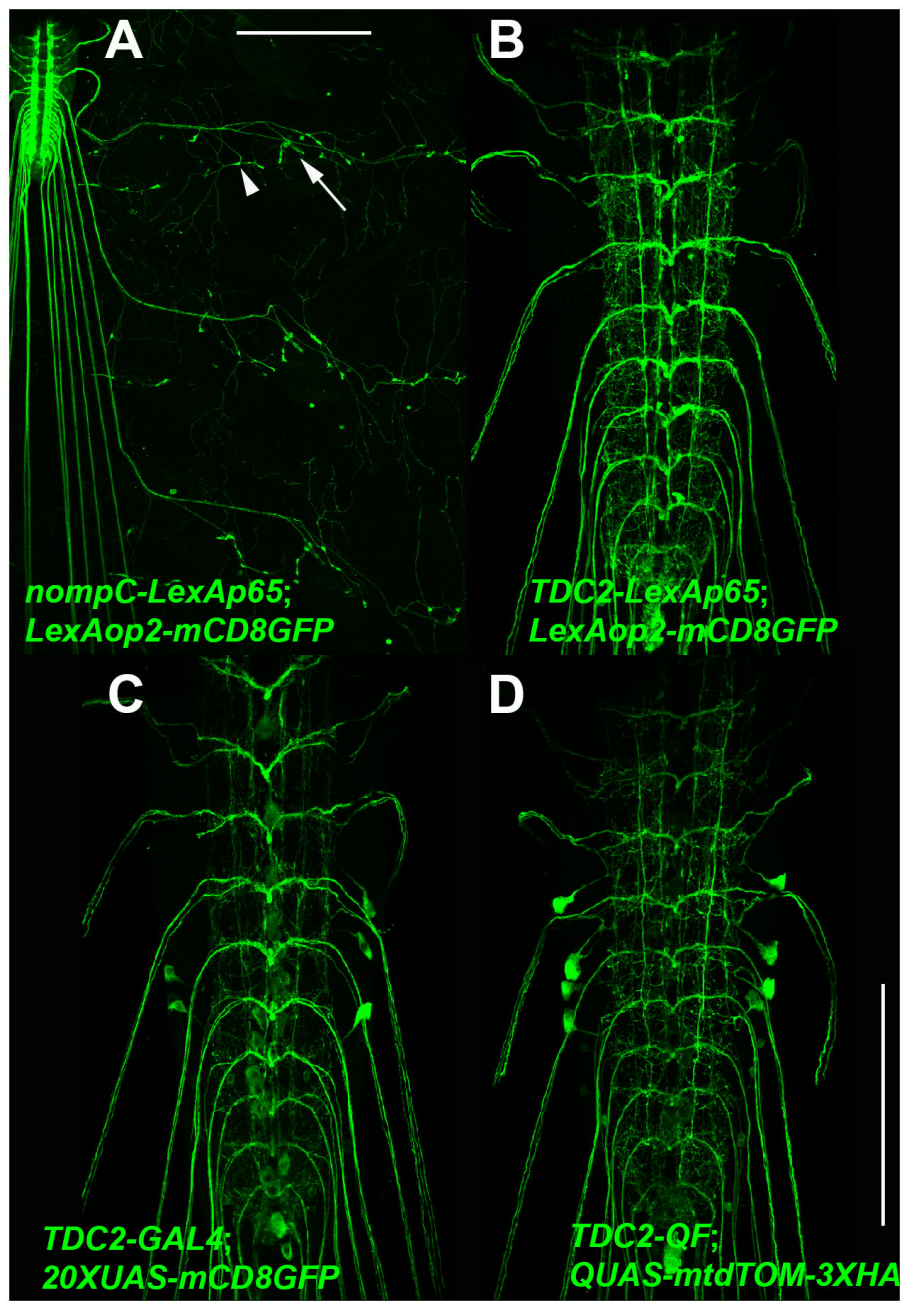
Functional demonstration of the L1-LexAp65-L4 Gateway MultiSite entry clone. Representative confocal images of third instar larva expression of (A) *yw*; *nompC-LexAp65*/*LexAop-mCD8GFP*; (B) *yw*; *TDC2-LexAp65*/*+*; *LexAop-mCD8GFP*/*+*; (C) *yw*; *20XUAS-mCD8GFP*/*+*;*TDC2-GAL4*/*+*; and (D) *yw*; *QUAS-mtdTOM-3XHA*/*+*; *TDC2-QF*/*+*. *nompC-LexAp65* expresses in class III larval sensory neurons and chordotonal organs. TDC2 drivers for LexAp65, GAL4, and QF express in presumptive tyraminergic and octopaminergic neurons. Slight differences in expression for TDC2 between the different binary transcription systems is likely due to distinct subcellular distribution of the different plasma membrane reporters. Primary antibodies were anti-GFP or anti-HA. Scale bars: (A) 200μm, (D) 100μm.

 To assess the functionality of the R4-LexAp65-R3 entry clone it was combined with L1-nompC 5’ Reg-L4 and L3-nompC 3’ Reg-L2 or L1-TDC2 5’ Reg’L4 and L3-TDC2 3’ Reg-L2 in separate three-fragment LR reactions to generate the expression clones nompC-LexAp65 and TDC2-LexAp65. The third instar larval expression pattern of nompC-LexAp65 is shown in Figure 5A using the reporter LexAop2-mCD8GFP. Similar to the previously reported nompC-GAL4 and nompC-QF drivers, nompC-LexAp65 exhibits expression specifically in class III larval sensory neurons (arrowhead) and chordotonal organs (arrow) [1], consistent with the known role of the *no mechanoreceptor potential C* (*nompC*) gene in mechanosensation [10,11]. 

 The third instar larval ventral nerve cord expression pattern of TDC2-LexAp65 is shown in Figure 5B using the reporter LexAop2-mCD8GFP. TDC2-LexAp65 exhibits a pattern highly reminiscent of the previously reported TDC2-GAL4 [12] in presumptive tyraminergic and octopaminergic neurons. The TDC2 gene is believed to decarboxylate tyrosine to convert it to the neurotransmitter tyramine [13]. These results demonstrate the functionality of the R4-LexAp65-R3, L1-TDC2 5’ Reg-L4, and L3-TDC2 3’ Reg-L2 entry clones. 

### Functional 13XLexAop2 Gateway MultiSite entry clones

 L1-13XLexAop2-R5 (compatible with two- and four-fragment Gateway MultiSite cloning) and L1-13XLexAop2-L4 entry clones (compatible with three-fragment Gateway Multisite cloning) were constructed. To assess the functionality of the L1-13XLexAop2-L4 entry clone it was combined with R4-GFP-R3 and L3-Rab3-L2, R4-mCherry-R3 and L3-Rab3-L2, or R4-n-syb-R3 and L3-4XmCherry-HA-L2 entry clones in separate three-fragment LR reactions to generate the expression clones 13XLexAop2-GFPRab3, 13XLexAop2-mCherryRab3, and 13XLexAop2-n-syb-4XmCherry-HA. 

**Figure 6:**
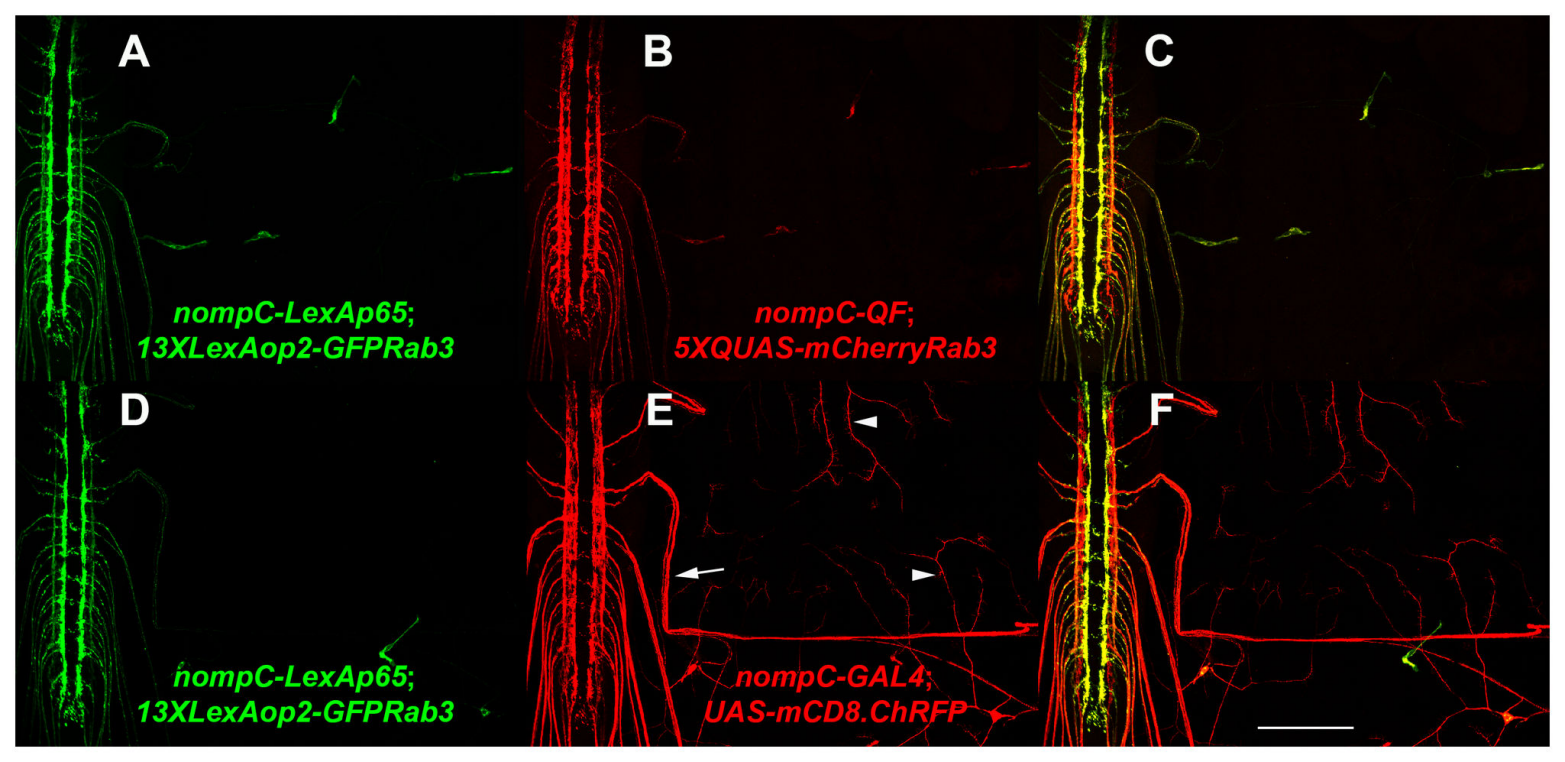
Functional demonstration of the L1-13XLexAop2-L4 Gateway MultiSite entry clone. Representative confocal images of third instar larval expression of (A-C) *yw*; *nompC-LexAp65*/*5XQUAS-mCherryRab3*; *nompC-QF*/*13XLexAop2-GFPRab3*; (D-F) *yw*; *nompC-LexAp65*/*nompC-GAL4*; *13XLexAop2-GFPRab3*/*UAS-mCD8*.*chRFP*. GFPRab3 expression via 13XLexAop2 shown in (A) and (D) preferentially accumulates in the sensory neuron presynaptic terminals similarly to mCherryRab3 shown in (B and C), and minimally in axons and dendrites like the plasma membrane marker mCD8mCherry shown in (E and F). Larvae were double-labeled with anti-GFP and anti-mCherry primary antibodies. The 13XLexAop2-GFPRab3 construct shown utilized the pDESThaw destination vector. Scale bar: 100μm.

 The third instar larval expression pattern of 13XLexAop2-GFPRab3 driven by nompC-LexAp65 is shown in Figure 6A as part of a double label experiment with the previously demonstrated synaptic vesicle reporter 5XQUAS-mCherryRab3 [1] driven with nompC-QF. The observed expression of GFP-Rab3 demonstrates the functionality of the L1-13XLexAop2-L4 entry clone. The localization of the vast majority of GFPRab3 to the presynaptic terminals of the chordotonal organs and class III sensory neurons in which the nompC-LexAp65 driver expresses indicates its utility as a synaptic vesicle marker. For comparison, 13XLexAop2-GFPRab3 expression driven by nompC-LexAp65 is shown in Figure 6D in a double label experiment with the plasma membrane reporter UAS-mCD8.ChRFP driven by nompC-GAL4 as shown in Figure 6E. While the mCD8.ChRFP plasma membrane marker shows significant localization to axons (arrow) and dendrites (arrowheads) the vast majority of GFPRab3 is restricted to the neuropil region of the larval ventral nerve cord where sensory neuron presynaptic terminals are located. 

 The reason for the reduced expression of nompC-LexA driven GFP-Rab3 in the lateral regions of the neuropil in Figures 6A and 6D as compared to that seen with nompC-QF driven mCherry-Rab3 in Figure 6B and nompC-GAL4 driven mCD8.ChRFP in Figure 6E is not known. Possible explanations include position effects of the insertion sites of the nompC-LexA driver or the LexAop2-GFP-Rab3 reporter, or that there is something intrinsic to the 13XLexAop2 operator sequence that results in reduced expression in the subset of neurons that innervate the lateral regions of the larval neuropil. 

**Figure 7:**
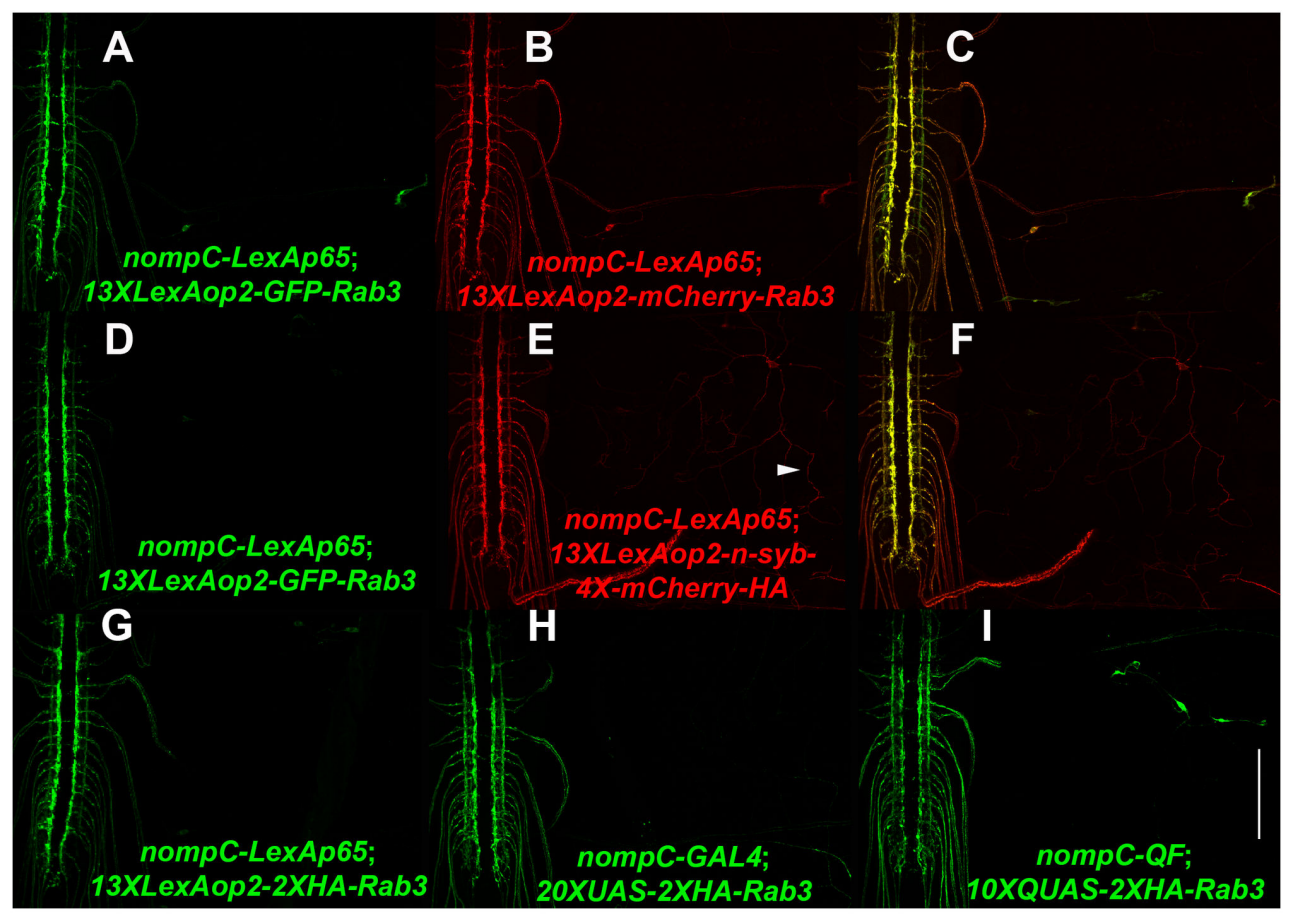
Functional demonstration of the L1-13XLexAop2-R5 Gateway MultiSite entry clone. Representative confocal images of third instar larval expression of (A-C) *yw; nompC-LexAp65*/*13XLexAop2-mCherryRab3*; *13XLexAop2-GFPRab3*/*+*; (D-F) *yw*; *nompC-LexAp65*/*13XLexAop2-n-sys-4XmCherry-HA*; *13XLexAop2-GFPRab3*; (G) *yw*; *nompC-LexAp65*/*13XLexAop2-HA-Rab3*; (H) *yw*; *20XUAS-HA-Rab3*/*+*; *nompC-GAL4*/*+*; (I) *yw*; *10XQUAS-HA-Rab3*/*+*; *nompC-QF*/*+*. mCherryRab3, n-syb-4XmCherry-HA, and 2XHA-Rab3 all localicalize prefentially to the presynaptic terminals of larval sensory neurons. Primary antibodies were anti-GFP, anti-mCherry, or anti-HA. Scale bar 100μm.

 Expression of the 13XLexAop2-mCherryRab3 and 13XLexAop2-n-syb-4X-mCherry-HA reporters in comparison to 13XLexAop2-GFPRab3 in double label experiments with the nompC-LexAp65 driver is shown in Figure 7. mCherryRab3 localization in Figure 7B is nearly identical to GFPRab3 expression in Figure 7A, thus demonstrating the utility of mCherryRab3 as a synaptic vesicle marker. The localization of n-syb-4X-mCherry-HA in Figure 7E also overlaps extensively with GFPRab3 in Figure 7D in sensory neuron presynaptic terminals, although weak expression of n-syb-4X-mCherry-HA can be seen in sensory neuron dendrites (arrowhead, 7E). The stronger preferential localization of GFPRab3 and mCherryRab3 to presynaptic terminals, as compared to n-syb-4X-mCherry-HA, indicates the superiority of the former as synaptic vesicle markers.

 To assess the functionality of the L1-13XLexAop2-R5 entry clone, it was combined with an L5-2XHA-Rab3-L2 entry clone in a two-fragment LR reaction to generate the 13XLexAop2-2XHA-Rab3 expression clone. The expression of 13XLexAop2-2XHA-Rab3 driven by nompC-LexA is shown in Figure 7G, thus demonstrating the functionality of both the L1-13XLexAop2-R5 and L5-2XHA-Rab3-L2 entry clones. 2XHA-Rab3 localizes preferentially to the presynaptic terminals of sensory neurons (compare 2XHA-Rab3 in 7G to GFPRab3 in 7A, D) indicating 2XHA-Rab3 is also a reliable synaptic vesicle marker. The lack of endogenous fluorescence from the HA tag, and the ability to detect it using anti-HA primary antibodies with secondary antibodies coupled to fluorophores of desired emission wavelength, make it compatible in multi-label experiments with other proteins having green and/or red fluorescent tags.

### Utility of Gateway MultiSite cloning

 An advantage of Gateway MultiSite cloning is the ease with which compatible entry clones can be partnered in LR reactions in desired combinations to assemble expression clones. The LexAp65 and LexAop2 entry clones reported here, in combination with the previously reported GAL4, QF, UAS, and QUAS entry clones [1], make a complete set of entry clones for generating drivers and reporters for all three *Drosophila* binary transcriptions systems [2,14,15] using Gateway MultiSite cloning. With Gateway MultiSite entry clones for all three binary transcription systems now available, generating the same driver or reporter for all three transcription systems is simple and efficient.

 As an example, an L1-TRH 5’ Reg-R5 entry clone was developed that contains regulatory sequences upstream of the tryptophan hydroxylase [TRH] gene that is believed to convert tryptophan to the serotonin neurotransmitter precursor 5-hydroxy-tryptophan [5-HT] [16]. This entry clone was combined in separate LR reactions with each of the existing entry clones L5-LexAp65-L2, L5-GAL4-L2, and L5-QF-L2 to generate the expression clones TRH-LexAp65, TRH-GAL4, and TRH-QF. 

**Figure 8:**
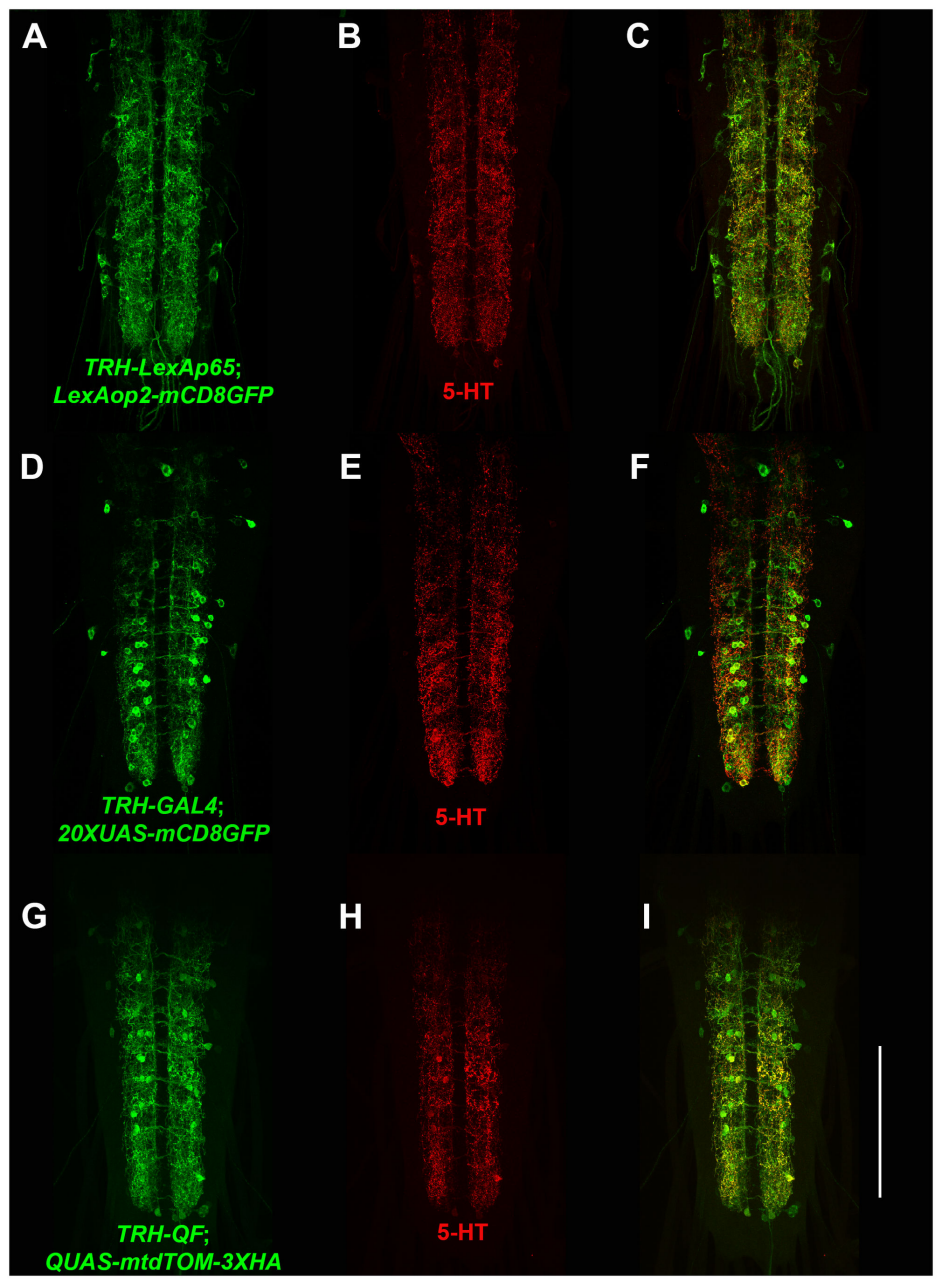
TRH drivers for the LexA, GAL4, and Q binary transcription systems express in 5-hydroxy-tryptophan neurons. Representative confocal images of third instar larval ventral nerve cord expression of (A-C) *yw*; *TRH-LexAp65*/*+*; *LexAop-mCD8GFP*/*+*; (D-F) *yw*; *TRH-GAL4*/*20XUAS-mCD8GFP*; and (G-I) *yw*; *TRH-QF*/*QUAS-mtdTOM-3XHA*. Ventral nerve cords were double-labeled with anti-5-HT and anti-GFP (A and D) or anti-HA (G) primary antibodies. All three TRH drivers accurately recapitulate expression specifically in serotonergic neurons. Scale bar: 100μm.

 Third instar larval ventral nerve cord expression of these expression clones using plasma membrane reporters is shown in Figure 8 along with the localization of 5-HT in double-label experiments. Comparison of the neuronal expression of TRH-LexAp65, TRH-GAL4, and TRH-QF as shown in Figures 8A, D, and G, respectively, with that of 5-HT as shown in Figures 8B, E, and H reveals complete overlap in neuronal expression between each of the drivers and 5-HT. It should be noted that subcellular localization of 5-HT and the plasma membrane localized reporters used in these experiments would not be expected to exhibit precise subcellular overlap because they distribute to distinct subcellular regions. The results of Figure 8 thus demonstrate the TRH drivers for all three binary transcription systems accurately recapitulate expression in serotonergic neurons of third instar larva.

 A similar approach was taken to generate TDC2 drivers for all three *Drosophila* binary transcription systems. TDC2-LexAp65 has already been described above, but TDC2-GAL4 and TDC2-QF drivers were also generated using the same L1-TDC2 5’ Reg-L4 and L1-TDC2 3’ Reg-L4 entry clones in combination with either R4-GAL4-R3 or R4-QF-R3 in separate LR reactions to assemble the expression clones TDC2-GAL4 and TDC2-QF. Third instar larval ventral nerve cord expression patterns of TDC2-GAL4 using the reporter 20XUAS-mCD8GFP and TDC2-QF using the reporter QUAS-mtdTOM-3XHA are shown in Figures 5C and 5D, respectively. As with TDC2-LexAp65, the TDC2-GAL4 and TDC-QF drivers exhibit expression patterns highly reminiscent of the previously characterized TDC2-GAL4 [12].

 The ease with which reporters for all three *Drosophila* transcription systems can be generated using Gateway MultiSite recombination cloning was demonstrated for 2XHA-Rab3 and Chr2 T159C-HA. The 13XLexAop2-2XHA-Rab3 synaptic vesicle reporter has already been described above, but the L5-2XHA-Rab3-L2 entry clone was also combined in separate LR reactions with the existing L1-20XUAS-R5 entry clone and a newly generated L1-10XQUAS-R5 entry clone to produce the expression clones 20XUAS-2XHA-Rab3 and 10XQUAS-2XHA-Rab3. Third instar larval expression of 20XUAS-2XHA-Rab3 driven by nompC-GAL4 and 10XQUAS-2XHA-Rab3 by nompC-QF are shown in Figures 7H and I, respectively. Similar to 13XLexAop2-2XHA-Rab3 (Figure 7G), they localize highly preferentially to the presynaptic terminals of larval sensory neurons (compare also to Figures 7A, D), thus demonstrating their utility as synaptic vesicle markers. Expression from 10XQUAS-2XHA-Rab3 shown in Figure 7I also demonstrates the functionality of the L1-10XQUAS-R5 entry clone.

**Figure 9:**
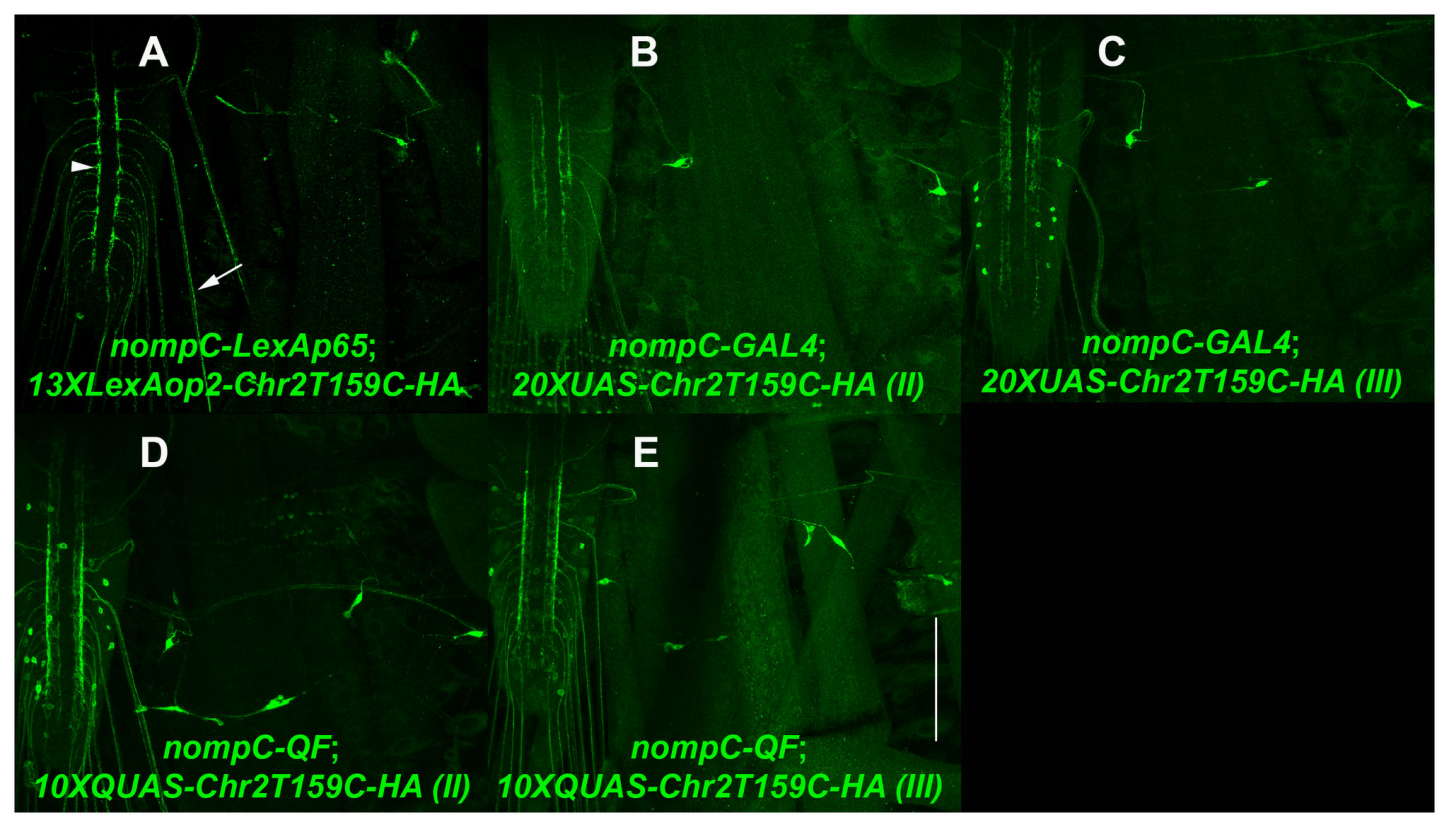
Expression of Channelrhodopsin T159C by the LexA, GAL4, and QF binary transcription systems. Representative confocal images of third instar larval expression of (A) *yw*; *nompC-LexAp65*/*+*; *13XLexAop2-Chr2T159C-HA*/*+*; (B) *yw*; *20XUAS-Chr2T159C-HA*/*+*; *nompC-GAL4*/*+*; (C) *yw*; *nompC-GAL4*/*+*; *20XUAS-Chr2T159C-HA*/*+*; (D) *yw*; *10XQUAS-Chr2T159C*/*+*; *nompC-QF*/*+*; (E) *yw*; *nompC-QF*/*+*; *10XQUAS-Chr2T159C-HA*/*+*. Chr2T159C-HA localizes preferentially to the cell bodies and pre-synaptic terminal of sensory neurons with weak localization to axons and almost no localization to dendrites. II-second chromosome insertion. III-third chromosome insertion. Anti-HA was used as the primary antibody. Scale bar: 100μm.

 Chr2 T159C-HA expression clones were also generated for all three *Drosophila* transcription systems. The T159C mutation of Chr2 was reported to result in nearly a doubling of stationary photocurrent amplitude in hippocampal neurons as compared to wildtype Chr2 [17] and would thus be expected to more potently induce action potentials in *Drosophila* neurons as compared to wildtype Chr2. An L5-Chr2 T159C-HA-L2 entry clone was generated (an HA epitope tag was added for assessing expression) and combined in separate LR reactions with L1-13XLexAop2-R5, L1-20XUAS-R5, or L1-10XQUAS-R5 entry clones to produce the expression clones 13XLexAop2-Chr2T159C-HA, 20XUAS-Chr2T159C-HA, and 10XQUAS-Chr2T159C-HA. Third instar larval expression of 13XLexAop2-Chr2T159C driven by nompC-LexAp65 is shown in Figure 9A. Chr2T159C-HA localizes predominantly to presynaptic terminals (arrowhead) with lower levels of expression in axons (arrow), and nearly undetectable levels in dendrites. 20XUAS-Chr2T159C-HA driven by nompC-GAL4 and 10XQUAS-Chr2T159C-HA driven by nompC-QF localize similarly as shown in Figures 9B, C and Figures 9D, E, respectively.

 To assess the functionality of these Chr2T159C-HA expression clones they were crossed to transcription system compatible nompC drivers and stimulated with blue light. Channelrhodopsin-mediated excitation of class III sensory neurons has been previously reported to induce “accordion” behavior whereby larvae contract their bodies to approximately half their fully extended body length [18]. The fly strains for all five Chr2T159C-HA expression clones shown in Figure 9 exhibited the expected “accordion” behavior upon stimulation with blue light (see videos S1-6 in Supporting Information) thus indicating all of them are functional. It should be noted that although Chr2 T159C results in nearly a doubling of photocurrent as compared to wildtype Chr2, and thus is presumably more potent at inducing behavioral responses than wildtype Chr2, it also has a significant afterpotential [17] that may make it unsuitable for experiments involving high frequency stimulation.

## Discussion

### New Gateway MultiSite destination vectors provide flexibility in transgene expression level

 The optimal level of transgene expression will vary for different experiments. For example, high expression levels are desirable in experiments using RNAi for gene silencing, or in other experiments expressing inhibitors of specific proteins or cellular processes, such as inhibitors of synaptic transmission like tetanus toxin or temperature-sensitive *shibire* [19,20]. In contrast, for experiments that involve specific targeting of a reporter to subcellular organelles such as mitochondria, ER, Golgi, or synaptic vesicles, lower levels of transgene expression might be desirable because high expression levels of subcellular organelle reporters may cause them to localize outside their desired location and thus lose their specificity. The pDESTsvaw and pDESTp10aw destination vectors make possible enhanced levels of transgene expression from Gateway MultiSite generated expression clones, each roughly doubling the level of transgene expression shown by the original *Drosophila* Gateway MultiSite vector pDESThaw. Greater differences in transgene expression levels would presumably result if both driver as well as the reporter were constructed in the pDESTsvaw or pDESTp10aw destination vectors, as compared to if both were constructed in pDESThaw. With these new Gateway MultiSite destination vectors now available, preference for transgene expression level can be dictated by choice of destination vector, depending on the needs of specific experiments. Further manipulation of transgene expression levels may also be possible in the future by the development of UAS, QUAS, or LexAop2 operator entry clones for Gateway MultiSite cloning with increased or decreased numbers of operator repeat sequences, or by including in entry clones 5’ UTR sequences known to modulate levels of transgene expression [3].

 An unexpected result from our quantitative and qualitative comparisons of the expression levels of GFP-Rab3 from the pDESTsvaw and pDESTp10aw destination vectors was that resulting expression levels were approximately equivalent. Our results contrast with a previous report in which the *p10* 3’ UTR was found to result in a more than10-fold enhancement of expression levels of cytoplasmic GFP as compared to the *SV40* 3’ UTR [4]. Reasons for this disparity are unknown, but could include that expression levels dictated by the different 3’ UTRs are gene or vector dependent, or that different methods of quantitation were used for the comparisons. 

### Gateway MultiSite cloning for the lexa binary transcription system

 The ability to independently express more than one transgene at a time is important for certain experiments such as optogenetic experiments where an excitatory channelrhodopsin is expressed in one set of neurons and a genetically-encoded calcium indicator in another [21], or GFP Reconstitution Across Synaptic Partners experiments where either of two fragments of GFP are expressed in distinct neuronal subsets [22]. Here we have reported the construction and functional demonstration of Gateway MultiSite entry clones for LexAp65 and 13XLexAop2. This will make possible construction of LexA system driver and reporter expression clones using Gateway MultiSite cloning for experiments requiring more than one binary transcription system. Such LexA system drivers and reporters can be used in conjunction with GAL4 or Q system drivers and reporters, or both, for experiments requiring independent control of expression of up to three transgenes simultaneously. 

### Harnessing the potential of Gateway MultiSite cloning

 A key feature that underlies the power of Gateway MultiSite cloning is the modular nature of entry clones. This modularity allows entry clones to be mixed and matched in compatible combinations in LR reactions to assemble expression clones. With a platform of entry clones now in place for all three binary transcription systems in use with *Drosophila*, including entry clones for the transcription factors GAL4, LexAp65, and QF, and their operators UAS, LexAop2, and QUAS, the potential for exploiting the advantages of Gateway MultiSite cloning has been further enhanced. 

 For any given entry clone containing regulatory DNA directing a specific temporal and spatial expression pattern, it is of only a slight amount of additional effort to generate a driver for all three transcription systems, as compared to the effort required to generate that driver for one system, due to the efficiency and reliability of Gateway MultiSite LR reactions [1]. This was demonstrated using entry clones containing regulatory DNA for the TDC2 and TRH neurotransmitter synthesis enzymes in combination with compatible entry clones for the GAL4, LexAp65, and QF transcription factors to generate driver expression clones for each transcription system for both TDC2 and TRH. 

 Similarly, with a given compatible protein-coding entry clone only minimal additional effort is required to generate reporters for all three transcription systems. This was demonstrated using entry clones containing 2XHA-Rab3 and Chr2T159C-HA in combination with compatible operator entry clones for UAS, LexAop2, and QUAS in LR reactions to generate reporter expression clones for all three transcription systems for both 2XHA-Rab3 and Chr2T159C-HA.

 For any existing regulatory DNA or protein-coding Gateway MultiSite entry clones, or those yet to be constructed, the capacity to easily and reliably generate driver and reporter expression clones for all three *Drosophila* binary transcription systems is now readily available. 

### Concluding remarks

 Here we presented and demonstrated the functionality of two new *Drosophila* Gateway MultiSite destination vectors, entry clones for the LexA binary transcription system, and numerous additional entry clones. These destination vectors and entry clones significantly enhance the utility of Gateway MultiSite cloning for generating expression clones for the *Drosophila* model system. Since Gateway MultiSite entry clones are model system-independent, the potential of Gateway MultiSite cloning is also enhanced for other genetic model organisms including mouse, zebrafish, and *C.elegans* pending only the development of suitable model system-specific destination vectors. 

## Materials and Methods

### Molecular biology

 BP and LR reactions were performed as previously described [1]. Templates for entry clones were as follows: L1-LexAp65-L4 and L1-LexAp65-R5⇒pBPLexA::p65Uw [3], L1-*13XLexAop2*-L4 and L1-*13XLexAop2*-R5⇒pJFRC19-*13XLexAop2*-IVS-myr::GFP [3], L5-2XHA-Rab3-L2⇒L3-Rab3-L2 [1], L5-Chr2 T159C-HA-L2⇒pCI-synapsin-Chr2 T159C [17], L1-10XQUAS-R5⇒ a gift plasmid from Chris Potter containing the 10XQUAS operator sequence. Inserts of all preceding entry clones were sequenced in their entirety to ensure no errors were introduced by PCR. The templates for L1-TDC2-L4, L3-TDC2-L2, L1-TRH-R5, and L1-n-syb-R5 were genomic DNA isolated from the strain *y; cn bw sp* [23]. Inserts of all entry clones containing genomic DNA were sequenced at both ends and exhibited the predicted restriction patterns. All other entry clones used herein were previously described [1]. 

 The pDESTsvaw destination vector was constructed by excising a 2.1kb *Hind III*/*Xba I* fragment from JFRC7 [3] and ligating it to a 1.7kb PCR fragment of the Gateway cassette to which *Hind III* and *Nhe I* sites were incorporated at the termini. The pDESTp10aw destination vector was constructed by excising a ~730bp *Xho I*/*Eco RI*-partial digest fragment containing the *SV40* 3’ UTR from pDESTsvaw, and ligating it to a ~700bp PCR fragment of the *p10* 3’ UTR to which *Xho I* and *Eco RI* sites were incorporated at the termini. The template for the *p10* 3’ UTR PCR was JFRC28 [4]. Both destination vectors contain *w*+ as a transformation marker and a *PhiC31* attB site for *PhiC31*-mediated transformation. 

 Fly stocks will be deposited at the Bloomington *Drosophila* stock center. Select plasmids will be deposited at Addgene.

### *Drosophila* stocks

 Flies were reared at 25C and raised on standard cornmeal/molasses media. Transgenic fly production was performed by Bestgene, Inc., Chino Hills, CA. Other fly strains used in this study besides those newly described here with Bloomington stock numbers in parentheses: *yw*; *QUAS-mtdTomato-3XHA* [15] (30004; 30005), *w*; *20XUAS-IVS-mCD8GFP* [3] (32194), *UAS-mCD8.ChRFP* (27391; 27392; F. Schnoorer, unpublished) LexAop-mCD8GFP [4], vGlut-LexA [24], nompC-GAL4 (36361, 36369) [1], nompC-QF (34346, 34349) [1], and 5XQUAS-mCherryRab3 (36353, 35364) [1].

### Immunohistochemistry

 Immunostaining was performed as previously described [1]. Images were collected on a Leica SP5 confocal microscope. Primary antibodies used in this study were rabbit anti-GFP Abfinity mAb (Invitrogen-Cat # G10362; 1:200), Rat anti-HA mAb 3F10 (Roche-Cat # 11 867 423 001; 1:200), Mouse anti-HA (Covance-Cat # MMS-101P; 1:500), Mouse anti-mCherry (Biorbyt-Cat # orb66657; 1:200), anti-5-HT (Thermo Scientific-Cat # MS-1431-S; 1:50). Secondary antibodies used in this study were goat anti-Rabbit Alexa Fluor 488 (Invitrogen-Cat # A-11034; 1:500), goat anti-mouse Alexa Fluor 488 (Invitrogen-Cat # A-11029; 1:500), goat anti-Mouse Alexa Fluor 568 (Invitrogen-Cat # A-11031; 1:500), goat anti-rat Alexa Fluor 488 (Invitrogen-Cat # A-11006; 1:500), and goat anti-rat Alexa Fluor 568 (Invitrogen-Cat # A-11077; 1:500).

### Image Quantitation

 Imarus x64 software v7.5.2 was used for intensity quantification in Figure 2D. Z-stacks were cropped to 512 X 1024 voxels and rendered as 3-dimensional surfaces using the Surpass feature. Uniform creation parameters were used across all images to subtract background, with a minimum of 15 voxels per surface. The Mean Intensity Maximum was thus averaged across all images for each genotype (two per animal, one on each side, n=5 animals for a total of 10 images per genotype). Values are displayed in Arbitrary Digitizer Units (ADU).

### Optogenetics

 Fly crosses for optogenetic behavioral experiments were cultured in vials on standard cornmeal/molasses media. A thin layer of yeast paste containing 1mm all-trans retinal (ATR) was added to the top of the food 24-48hrs before an experiment. Wandering third instar larva were used for all optogenetic experiments. Light stimulation was provided by a manually controlled light guide attached to a Sutter Lambda DG-4 wavelength switcher with a 475-495nm single bandpass filter. 

## Supporting Information

Video S1 **Accordion behavior is not elicited by negative control (no all-trans retinal) larva expressing Chr2 T159C-HA in class III sensory neurons and chordotonal organs upon blue light stimulation.** Genotype: *yw*; *nompC-GAL4*/+; *20XUAS-Chr2* *T59C-HA*/+. The presence of the blue rectangle indicates larvae are being stimulated with blue light.
(MP4)

Video S2 **Accordion behavior is elicited by larva expressing Chr2 T159C-HA under 13XLexAop2 control in class III sensory neurons and chordotonal organs upon blue light stimulation.** Genotype: *yw*; *nompC-LexAp65*/*13XLexAop2-Chr2* *T159C-HA*. The presence of the blue rectangle indicates larvae are being stimulated with blue light.
(MP4)

Video S3 **Accordion behavior is elicited by larva expressing Chr2 T159C-HA under 20XUAS control in class III sensory neurons and chordotonal organs upon blue light stimulation.** Genotype: *yw*; *20XUAS-Chr2* *T159C-HA*/*+*; *nompC-GAL4*/*+*. The presence of the blue rectangle indicates larvae are being stimulated with blue light.
(MP4)

Video S4 **Accordion behavior is elicited by larva expressing Chr2 T159C-HA under 20XUAS control in class III sensory neurons and chordotonal organs upon blue light stimulation**. Genotype: *yw*; *nompC-GAL4*/*+*; *20XUAS-Chr2* *T159C-HA*/*+*. The presence of the blue rectangle indicates larvae are being stimulated with blue light.
(MP4)

Video S5 **Accordion behavior is elicited by larva expressing Chr2 T159C-HA under 10XQUAS control in class III sensory neurons and chordotonal organs upon blue light stimulation.** Genotype: *yw*; *10XQUAS-Chr2* *T159C-HA*/*+*; *nompC-QF*/*+*. The presence of the blue rectangle indicates larvae are being stimulated with blue light.
(MP4)

Video S6 **Accordion behavior is elicited by larva expressing Chr2 T159C-HA under 10XQUAS control in class III sensory neurons and chordotonal organs upon blue light stimulation.** Genotype: *yw*; *nompC-QF*/*+*; *10XQUAS-Chr2* *T159C-HA*/*+*. The presence of the blue rectangle indicates larvae are being stimulated with blue light.
(MP4)
